# Preparation and Characterization of Highly Porous Polyacrylonitrile Electrospun Nanofibers Using Lignin as Soft Template via Selective Chemical Dissolution Technique

**DOI:** 10.3390/polym13223938

**Published:** 2021-11-15

**Authors:** Mohd Adib Tajuddin Ahmad, Norizah Abdul Rahman

**Affiliations:** 1 Department of Chemistry, Faculty of Science, Universiti Putra Malaysia, Serdang 43400, Selangor, Malaysia; adibataupm@gmail.com; 2 Materials Synthesis and Characterization Laboratory, Institute of Advanced Technology, Universiti Putra Malaysia, Serdang 43400, Selangor, Malaysia

**Keywords:** polyacrylonitrile, lignin, electrospinning, selective chemical dissolution, porous nanofibers, nanofibers, soft template

## Abstract

In this study, polyacrylonitrile (PAN) was mixed with a renewable polymer, lignin, to produce electrospun nanofibers by using an electrospinning technique. Lignin was utilized as a soft template that was removed from the nanofibers by using a selective dissolution technique to create porous PAN nanofibers. These nanofibers were characterized with Fourier transform infrared (FTIR), field emission scanning electron microscopy (FESEM), thermogravimetry analysis (TGA), X-ray diffraction (XRD), and Brunauer-Emmett-Teller (BET) to study their properties and morphology. The results showed that lignin can be homogeneously mixed into the PAN solution and successfully electrospun into nanofibers. FESEM results showed a strong relationship between the PAN: lignin ratio and the diameter of the electrospun fibers. Lignin was successfully removed from electrospun nanofibers by a selective chemical dissolution technique, which resulted in roughness and porousness on the surface of the nanofibers. Based on the BET result, the specific surface area of the PAN/lignin nanofibers was more than doubled following the removal of lignin compared to PAN nanofibers. The highest specific surface area of nanofibers after selective chemical dissolution was found at an 8:2 ratio of PAN/lignin, which was 32.42 m^2^g^−1^ with an average pore diameter of 5.02 nm. The diameter of electrospun nanofibers was also slightly reduced after selective chemical dissolution. Porous PAN nanofibers can be seen as the precursors to the production of highly porous carbon nanofibers.

## 1. Introduction

In recent years, nanotechnology has become a promising technology for the future. There are many types of nanomaterials, such as nanoparticles, nanofibers, nanotubes, nanowires, and quantum dots, that have garnered extensive attention due to their unique properties and characteristics [1]. Among these nanomaterials, nanofibers are receiving significant attention from researchers due to their holding properties such as high porosity, a high surface area and controllable morphology, and high chemical and thermal stability [2,3]. Several techniques can be used to produce nanofibers, such as template-assisted synthesis [4], chemical vapor synthesis [5], and electrospinning.

At present, electrospinning techniques have received interest as they are economical and suited to various types of material [6], good for controlling fiber morphology [7], and easy to operate [8]. A high-voltage power supply, a conductive collector, and a spinneret are the important, basic forms of equipment that are needed for the electrospinning process. Electrospinning involves an electrohydrodynamic process, during which a high voltage is applied to a polymer droplet; this is designed to reduce the surface tension of the polymer droplet and transform it into a pointed shape. Increasing the voltage supply will trigger the formation of a Taylor cone and a jet from the tip of needle. The solvent evaporates as the jet travels to the collector, and to prevent the jet from forming into droplets (electrospraying), sufficient entanglement of the polymer occurs [9]. An electrospinning technique is a distinctive way to produce nanofibers from various types of polymers with a diameter ranging from micrometer to nanometer, and to produce a higher surface area than those obtained from conventional spinning processes [10]. Thus, electrospinning is the best tool for the production of polymer nanofibers as it is economical and the simplest method [11].

Recently, a significant number of studies that have used nanofibers as an adsorbent for the removal of pollutants from aqueous solutions, such as drugs [12,13] and heavy metals [14,15,16,17], has been reported. For the application of nanofibers as adsorbent, the adsorption process is crucial. During this process, the adsorbate molecules are transported into the surface of the absorbent. The internal mass transfer is transported to the inner surface of the porous structure from the outer surface of the adsorbent [18]. Targeted contaminant removal depends on the pore size of the adsorbent [19]. The pores of the nanofibers are made up of several categories: micropores (<2 nm), mesopores (2–50 nm), and macropores (>50 nm). The main part of the adsorption process occurs with mesopores and micropores [20].

Polyacrylonitrile (PAN) is a polymer usually used as the precursor to the formation of carbon fibers due to its excellent mechanical strength, low flammability, good thermal stability and chemical resistance [21], such as PAN-based carbon fiber electrodes for energy storage application [22,23] and for thermal materials [24]. It is a homo-polymer made up of acrylonitrile. PAN contains a nitrile group, known as a polar group, that helps it to be soluble in a highly polar solvent, and helps increase its melting points. The interaction of the nitrile group and the polymeric backbone of the polymer hinder the molecules of PAN carbon fibers to align in a particular direction. Dimethylformamide (DMF) is the most used solvent for dissolving PAN to make nanofibers; the solvent helps to reduce the interaction between the nitrile groups, and helps provide a better orientation of the produced polymer chain [25].

Lignin is among the most promising biorenewable raw materials, and it is a natural polymer that acts as a partial matrix within the structure of plants and trees. It is one of the main elements of wood and is the second most abundant natural polymer. Lignin is a highly branched macromolecular network structure, with an aromatic nature and complex compositions. Furthermore, lignin has a lack of cytotoxicity, which proves that lignin is a type of biomaterial [26]. Industries such as the pulp and paper industry are the main sources of lignin as a by-product and of second-generation bioethanol facilities derived from lignocellulosic sources [27]. The structure of lignin is composed of phenylpropene units (monolignols) of the hydroxyl group, but the plant source and its isolation process impact lignin complex configuration (Duval and Lawoko, 2014). Lignin has some advantages, such as a high carbon content, its suitability with various chemicals, good thermal stability, and its low cost and biodegradability [28]; however, there are some limitations, such as the relative heterogeneity of its structure and difficulties in its processing that still hinder its more widespread use [29]. Lignin has received interest among researchers as a potential low-cost carbon fiber precursor because it is easy to obtain and has a high carbon content.

The selective chemical dissolution technique is one of the processes to produce a porous structure and higher surface area in a polymer. For porous polymer nanofibers, this can be achieved through the electrospinning of polymer blend solutions to form a nanofiber, followed by the selective chemical dissolution process which involves the removal of one component using an appropriate solvent that only dissolves a certain polymer in the blend’s nanofibers. Another alternative is the addition of additives such as salts or nanoparticles in the polymer solution that are used as a template, followed by their removal by post-electrospinning processes; the elimination of the template is another method to create a porous structure [30]. In a study by Chen et al. [31], silk fibroin/poly (L-lactic acid) (SF/PLA) fibers were produced by an electrospinning technique. The silk fibroin was removed from the fibers by dissolving the fibers in chloroform for up to 1 h. After the selective chemical dissolution process, the fibers showed a porous structure with irregular shapes [31]. In another study, Kim et al. [23] used electrospinning to produce poly(vinyl alcohol) (PVA) and PAN blend fibers. Either PVA or PAN can be removed from the nanofibers. PVA was successfully removed from the fibers by dipping them in hot water and acetic acid, while PAN was eliminated from the fibers using DMF treatment. Both results showed the surface diameter of the electrospun fibers as decreasing, and the surface of the fibers revealed grooves along the axis and appeared rougher [32].

In this study, alkali lignin, with a low sulfonate content, was incorporated with PAN to produce nanofibers. A renewable polymer, lignin was used as a soft template to create a porous and rougher surface for the nanofibers through the selective chemical dissolution technique.

## 2. Materials and Methods

### 2.1. Reagents

The materials used in this experiment were alkali lignin (low sulfonate content) and polyacrylonitrile (PAN) (average M_w_ = 150,000), and were purchased from Sigma-Aldrich (Merck Group, St. Louis, MO, USA). *N,N*-dimethylformamide (DMF) was obtained from R&M Chemicals (Ever Gainful ENT., Ara Damansara, Selangor, Malaysia). De-ionized water was used throughout the experiment.

### 2.2. Preparation of Electrospun Nanofibers

PAN nanofibers were prepared from a PAN solution dissolved in DMF with a concentration of 7.5%. A 5 mL syringe was loaded with the PAN solution, and a 0.8 mm inner diameter needle was attached with the syringe. The distance between the tip of the needle and the aluminum foil collector was fixed at 10 cm. The electrospinning was conducted using 18 kV voltage with a 2 mL/h flow rate. The nanofibers produced were collected on the aluminum foil and characterized. Following this, the PAN/lignin nanofibers were prepared in the same way as the PAN nanofibers. The ratios of PAN/lignin were varied: 9:1, 8:2, 7:3, and 6:4.

### 2.3. Selective Chemical Dissolution Technique

Lignin is a water-soluble polymer, while PAN is insoluble in water. Thus, PAN/lignin nanofibers were immersed in de-ionized water at 60 °C for 30 min to eliminate lignin from the electrospun nanofibers, and then the nanofibers were dried in a convection oven for 3 h at 60 °C and sent for characterization. This procedure was repeated for the PAN nanofibers for comparison purposes.

### 2.4. Characterizations

FTIR analyses of the samples were conducted to determine the functional groups using an attenuated total reflectance-Fourier transform infrared spectroscopy (ATR–FTIR) spectrometer (Perkin Elmer Spectrum RXI, Waltham, MA, USA). The wavelength used to examine the functional groups ranged from 280 to 4000 cm^−1^. The thermal properties of the samples were determined using thermal gravimetric analysis (TGA) (Mettler Toledo Thermogravimetric Model TGA/SDTA, Columbus, OH, USA). The samples were heated from 50 °C to 600 °C, with a heating rate of 10 °C/min under a nitrogen atmosphere. Field emission scanning electron microscopy (FESEM) (FEI NOVA NANOSEM 230, Hillsboro, OR, USA) was used to examine the morphologies of the nanofibers. A stub was taped with carbon tape and the samples were placed onto it. The samples were then sputtered with a thin layer of gold. The average fiber diameter was determined by using ImageJ Software, with 200 diameter readings taken. X-ray diffraction (XRD) (Shimadzu Model XRD-6000, Kyoto, Japan) analyses of the samples occurred to identify the crystalline phase, where the scan started from 2θ range and from 2° to 60°, with a scanning rate of 2°/min. The surface area and porosity of the prepared nanofiber were determined by N_2_ adsorption measurements at 77K using BELSORP Mini II. The surface characterization of the nanofibers was obtained using the Brunauer-Emmet-Teller (BET Micromeritics Instrument Corporation, Model-3Flex, Norcross, GA, USA) method.

## 3. Results and Discussion

### 3.1. Fourier Transform Infrared Spectra (FTIR) Analysis

**Figure 1:**
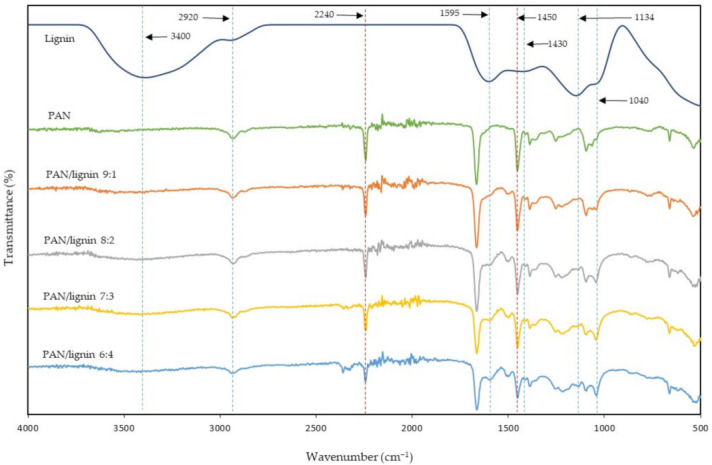
FTIR spectra of the PAN/lignin nanofibers after selective chemical dissolution compared to PAN and lignin.

ATR-FTIR technique was used to analyze the PAN, lignin, and PAN/lignin nanofibers before and after selective chemical dissolution. Figure 1 shows the FTIR spectra of PAN, lignin, and PAN/lignin nanofibers. The ratio of PAN: lignin was varied at 9:1, 8:2, 7:3, and 6:4 to study the effect of different ratios on the fabricated PAN/lignin nanofibers. The IR spectrum of lignin shows the major lignin bands at 3400 cm^−1^ (O–H stretching), 1595 cm^−1^ (aromatic skeletal vibration), 1430 cm^−1^ (C–C stretching with C–H deformation), 1134 cm^−1^ (C–H deformation), and 1040 cm^−1^ (C–O deformation) [33]. In Figure 1, it can be seen that the higher the lignin content in the fiber, the higher the intensity of the lignin peaks at 1595 cm^−1^, 1134 cm^−1^, and 1040 cm^−1^. A small peak at 2920 cm^−1^ indicates a stretching of the alkyl C–H bond and a stretching of the intermolecular hydrogen bonding of the O–H group, which is associated with the hydroxyl group in the lignin [34]. A strong peak at 2240 cm^−1^ indicates the C≡N group of PAN; the intensity of the peak was decreased as the ratio of PAN decreased. A peak at around 1450 cm^−1^ corresponds to the bending of the CH_2_ scissoring of PAN [35]. The intensity of the peak at 1450 cm^−1^ was reduced when the ratio of lignin increased. As can be seen in Figure 1, the main peak position of PAN did not shift for all the nanofibers, demonstrates that the blending of PAN and lignin only involves physical blending.

**Figure 2:**
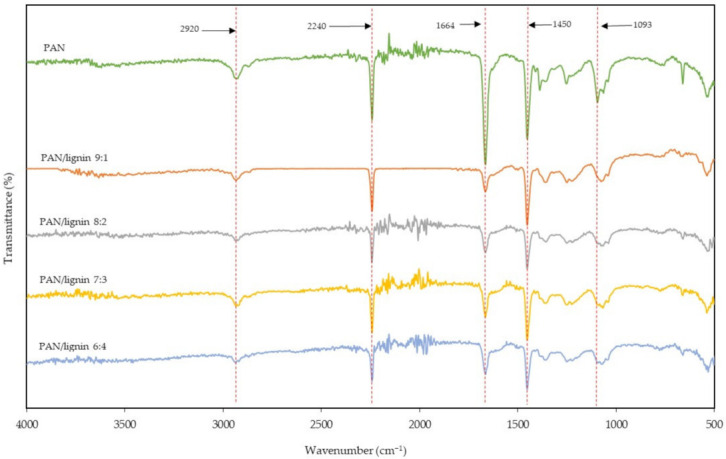
FTIR spectra of pure PAN and PAN/lignin nanofibers after selective chemical dissolution process.

The FTIR spectra of the PAN/lignin nanofibers after the selective chemical dissolution process when compared to PAN and lignin is shown in Figure 2. The IR spectrum of PAN shows peaks at around 2920, 2240, 1664, and 1450 cm^−1^. These peaks indicate the alkyl C–H bond stretching, C≡N stretching, C≡N stretching, and CH_2_ bending, respectively [36,37,38]. Selective chemical dissolution was performed to remove the lignin from the nanofibers. The results showed that, for the nanofibers after treatment, most IR peaks in PAN/lignin nanofibers exhibited mainly belong to PAN. In comparison, the peaks that belong to lignin, such as the peaks at around 1595 cm^−1^ and 1040 cm^−1^, were reduced significantly. This is because the lignin content in the nanofibers was successfully removed through the selective chemical dissolution process.

### 3.2. Field Emission Scanning Electron Microscope (FESEM) Analysis

**Figure 3:**
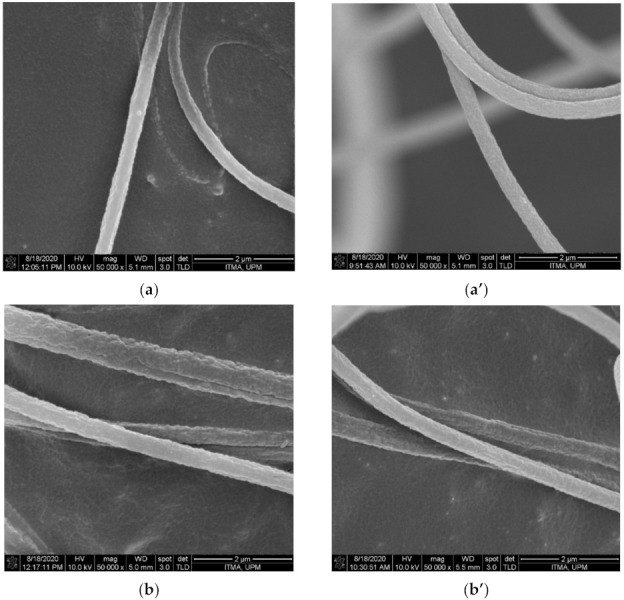

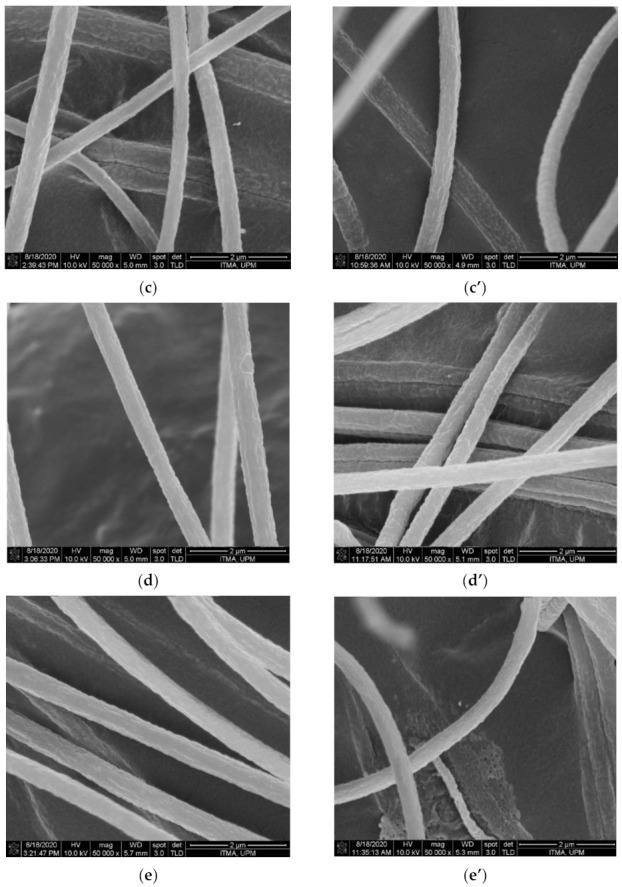
FESEM micrograph of nanofibers with various PAN: lignin ratios (**a**) PAN, (**b**) 9:1, (**c**) 8:2, (**d**) 7:3, (**e**) 6:4 ratio before selective chemical dissolution, and (**a’**) PAN, (**b**’) 9:1, (**c’**) 8:2, (**d’**) 7:3, and (**e’**) 6:4 ratio after selective chemical dissolution.

**Figure 4:**
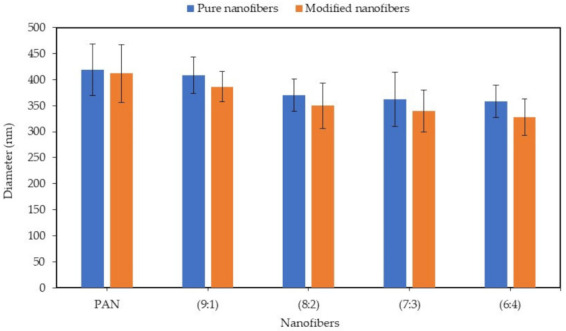
Graph of the diameter of nanofibers before (pure nanofibers) and after (modified nanofibers) selective chemical dissolution.

The morphology of the nanofibers was investigated using the FESEM technique. Figure 3 and Figure 4 show FESEM images and the average diameter of the PAN/lignin nanofibers against the various ratios of PAN: lignin nanofibers, respectively. The PAN nanofibers did not contain lignin, but underwent a selective chemical dissolution process for comparison purposes. From the FESEM images in Figure 3, it can be observed that before selective chemical dissolution, the nanofibers’ surface is smoother than after selective chemical dissolution. However, it can be seen that some pores and rough surfaces appear on the nanofibers before selective chemical dissolution, possibly due to the effect of rapid solvent evaporation during electrospinning and drying proses. After selective chemical dissolution, the surface nanofibers become rougher and more porous due to the removal of lignin during hot de-ionized water treatment. The average diameter of nanofibers before and after selective chemical dissolution decreased when the content of lignin increased from a ratio of 9:1 to 6:4. The average diameter of PAN and PAN/lignin nanofibers at ratios of 9:1, 8:2, 7:3, and 6:4 before selective chemical dissolution was 419 ± 49 nm, 409 ± 35 nm, 371 ± 31 nm, 362 ± 52 nm, and 358 ± 31 nm, respectively. When the content of lignin increased, the average diameter of nanofibers decreased. This is due to a decrease in the viscosity of the polymer solution that produces a lower fiber diameter, as mentioned earlier. This is due to the lower molecular weight and higher polydispersity index of lignin compared to PAN [39]. The average diameter of PAN and PAN/lignin nanofibers after dissolution was slightly decreased compared with before selective chemical dissolution; 412 ± 55 nm, 386 ± 29 nm, 349 ± 44 nm, 340 ± 40 nm, and 327 ± 35 nm for PAN and PAN/lignin with ratios of 9:1, 8:2, 7:3, and 6:4, respectively. The FESEM images show that all the nanofibers before and after dissolution were bead-free, and that the nanofibers had a non-uniform, porous, and rough surface. This proves that, by using the selective chemical dissolution technique, lignin could be successfully removed from nanofibers and could produce a rough surface and cause a high porosity of PAN/lignin nanofibers, providing a higher surface area. However, FESEM images only are insufficient to prove the higher surface area of nanofibers after selective chemical dissolution. These results can be supported by Brunauer-Emmett-Teller (BET) analysis, which will be discussed later in Section 3.5.

### 3.3. Thermal Analysis

**Figure 5:**
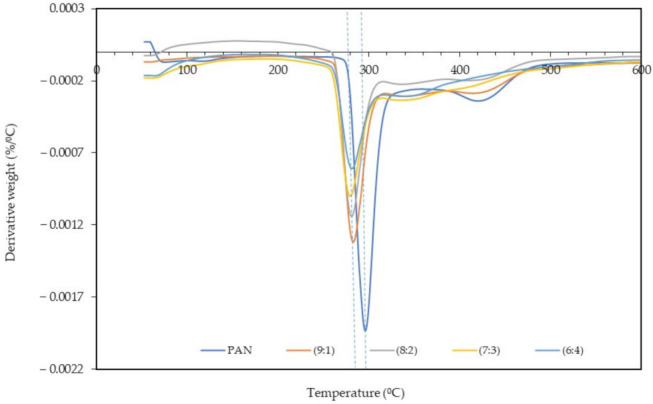
DTG curve of nanofibers before selective chemical dissolution.

**Figure 6:**
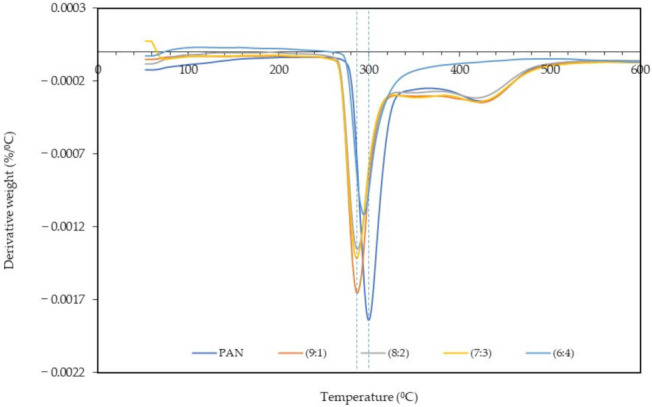
DTG curve of nanofibers after selective chemical dissolution.

The derivative thermogravimetry (DTG) and TGA analyses of the PAN: lignin nanofibers before and after selective chemical dissolution was carried out to study the thermal stability of the samples. Figure 5 and Figure 6 show the DTG thermogram at different ratios of PAN: lignin nanofibers, from 50 °C to 600 °C. TGA thermograms of nanofibers before and after selective dissolution are shown in Supplementary Materials (Figures S1 and S2). The DTG and TGA thermograms of nanofibers both before and after selective chemical dissolution show a similar trend. The first weight loss can be observed at 50–120 °C and is due to the loss of water molecules that are bound to the nanofibers. TGA thermograms of the nanofibers (Figures S1 and S2) show that all the nanofibers gradually decomposed at a higher temperature range. From DTG and TGA thermograms, the main degradation began at 275 °C for PAN nanofibers, attributed to the pyrolysis of the nanofibers [40]. The addition of lignin to the nanofibers (PAN/lignin nanofibers) lowered the onset degradation temperature of the fibers (~260 °C) compared to PAN nanofibers; however, there was no significant difference between the various ratios of lignin in the fibers. The addition of lignin slightly reduced the thermal stability of the nanofibers. When comparing the results before and after selective chemical dissolution, the onset degradation temperature of the PAN/lignin nanofibers was slightly higher than before selective chemical dissolution, possibly due to the removal of lignin and because the nanofibers are mostly composed of PAN, which has a higher thermal stability than lignin. However, after selective dissolution, the onset temperature of the PAN/lignin nanofibers was still slightly lower than PAN nanofibers (Figure 6). It is noticeable that the PAN/lignin (8:2) in Figure S1 and the PAN/lignin (6:4) in Figure S2 show that weight loss exceeded 100%. The possible explanation for this is that the PAN fiber can have a reaction with nitrogen under thermal treatment [41] with a resulting weight increase of over 100%.

From DTG thermograms, a sharp peak corresponding to a maximum decomposition temperature of PAN nanofibers is observed at 296 °C (Figure 5) and 299 °C (Figure 6) for before and after selective chemical dissolution, respectively. The maximum degradation temperature of the PAN/lignin nanofibers before selective chemical dissolution occurred at a temperature lower than PAN, which is 282 °C, and after the removal of lignin, the maximum degradation temperature shifted to a higher temperature in the range of 287–295 °C. This is because the removal of lignin caused the nanofibers to be composed of more PAN, and provided the nanofiber with higher thermal stability. These results also prove that the removal of lignin after de-ionized water treatment successfully occurred. It is further confirmed that the incorporation of lignin reduces the thermal stability of nanofibers.

### 3.4. X-ray Diffractometer (XRD) Analysis

**Figure 7:**
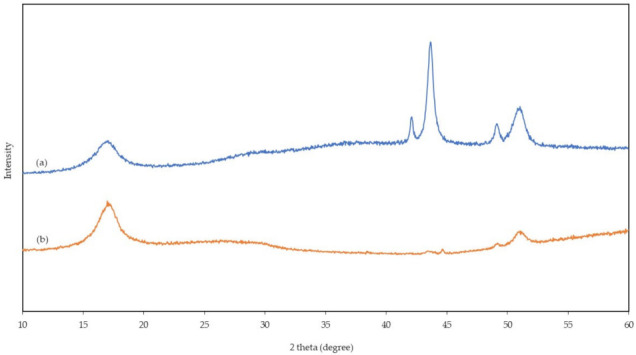
XRD spectra of (**a**) 7:3 PAN/lignin nanofiber and (**b**) 7:3 PAN/lignin nanofibers after selective chemical dissolution.

Figure 7 shows the sample result of XRD analyses carried out to study the crystallinity of nanofibers before (pure 7:3 PAN/lignin nanofibers) and after (modified 7:3 PAN/lignin nanofibers) selective chemical dissolution. The XRD diffraction pattern of the nanofibers before and after selective chemical dissolution shows a broad peak at around 17°, which can be ascribed to (100) crystallographic planes of PAN due to their amorphous nature [14,42]. Nevertheless, the intensities of peaks at 17° were increased with the removal of lignin content in PAN-based nanofibers after selective chemical dissolution. Crystallinity decreased with the higher lignin content as lignin is a biopolymer consisting of amorphous phenyl propylene [43]. The XRD patterns of the pure 7:3 PAN/lignin nanofibers show characteristic peaks of 2 theta located at 42°, 44°,49°, and 51° that may be due to the blend of PAN with lignin. Once modified, intensity of all these peaks were reduced due to lignin removal.

### 3.5. BET Analysis

The most frequent approach to the determination of specific surface areas of porous materials is the Brunauer-Emmett-Teller (BET) approach. The pore information of the porous nanofibers, including the BET-specific surface area and the microporous and mesoporous volumes, is summarized in Table 1. As seen in Table 1, the specific surface area of PAN: lignin nanofibers after selective chemical dissolution are 16.17 m^2^g^−1^, 20.94 m^2^g^−1^, 32.42 m^2^g^−1^, 29.13 m^2^g^−1^, and 28.15 m^2^g^−1^ for PAN, with 9:1, 8:2, 7:3, and 6:4 ratios, respectively. The PAN: lignin nanofibers of 8:2 ratio showed the highest BET surface area (32.42 m^2^g^−1^), followed by 7:3, 6:4, 9:1, and PAN. This shows an increase in specific surface area of up to 50% compared to PAN nanofibers after the removal of lignin. The specific surface area ratio from 7:3 to 6:3 is a slight decrease from 8:2, possibly due to the excess lignin addition to the nanofibers, which creates a bigger pore diameter and consequently reduces the specific surface area. In this study, the increase in the specific surface area is greater than reported elsewhere in the literature. For example, Ji and co-workers [44] studied the effect of PAN and silica nanoparticles using a selective chemical dissolution process. The silica component was removed from silica/PAN composite fibers, and the surface area of the nanofibers was increased by only 20%.

**Figure 8:**
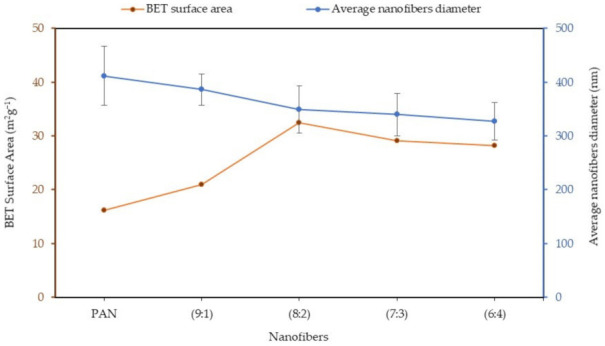
Graph of various ratios of PAN/lignin nanofibers, with BET surface area and average diameter.

The interrelationship between the average diameter and the BET-specific surface area of nanofibers as a function of a ratio of PAN/lignin nanofibers after selective chemical dissolution is shown in Figure 8. From the graph, it can be seen that the higher the composition of lignin in the nanofibers, the lower the average diameter of the fiber and the higher the BET-specific surface area. The optimum surface area of the porous nanofibers can be obtained by only a 20% incorporation of lignin fibers with a very small pore diameter (5.02 nm) and pore volume (0.1974 m^3^g^−1^).

## 4. Conclusions

The highly porous and specific surface area of PAN/lignin electrospun nanofibers was successfully prepared by using lignin as a soft template and through a selective chemical dissolution technique. The morphology showed a significant change in the surface of the nanofibers before and after selective chemical dissolution technique. After selective chemical dissolution, the nanofibers’ surface appeared to be rougher and slightly smaller in average fiber diameter than before selective chemical dissolution. The BET analysis showed a significant increase in specific surface area after the addition of lignin to the PAN/lignin nanofibers, and the pore diameter was varied with the various ratios of lignin in the nanofibers. The optimum specific surface area of PAN/lignin nanofibers was 8:2 ratio, which is 32.42 m^2^g^−1^ with a pore diameter of 5.02 nm. Additional lignin not only impacts the morphology of the nanofibers, but also the fiber diameter. The higher the ratio of lignin added, the smaller the average fiber diameter. This study showed that lignin and PAN can be homogenously blended and utilized to produce the high surface area and porosity of PAN nanofibers using a selective chemical dissolution technique. This can be used in an extensive variety of applications, for instance, as a precursor to highly porous carbon nanofibers.

## Figures and Tables

**Table 1:** Specific surface areas (S_BET_), pore parameters of PAN/lignin nanofibers after selective chemical dissolution with different PAN: lignin ratios.

	PAN	9:1	8:2	7:3	6:4
Specific surface area, S_BET/_m^2^g^−1^	16.17	20.94	32.42	29.13	28.15
Pore volume, V_total_/m^3^g^−1^	0.2360	0.2371	0.1974	0.1800	0.2970
Average pore diameter/nm	5.16	5.13	5.02	5.34	5.08

## Data Availability

Not applicable.

## Supplementary Materials

The following are available online at https://www.mdpi.com/article/10.3390/polym13223938/s1, Figure S1: TGA curve of nanofibers before selective chemical dissolution, Figure S2: TGA curve of nanofibers after selective chemical dissolution.
