# Supplementary material for: Preparation and Characterization of Highly Porous Polyacrylonitrile Electrospun Nanofibers Using Lignin as Soft Template via Selective Chemical Dissolution Technique
Source: Polymers (Basel). 2021 Nov 15;13(22):3938. doi: 10.3390/polym13223938 (PMC8617785; doi:10.3390/polym13223938)
Supplement: Supplementary file 1 [file polymers-13-03938-s001.zip › polymers-1402998-supplementary.pdf]

Supplementary Materials:

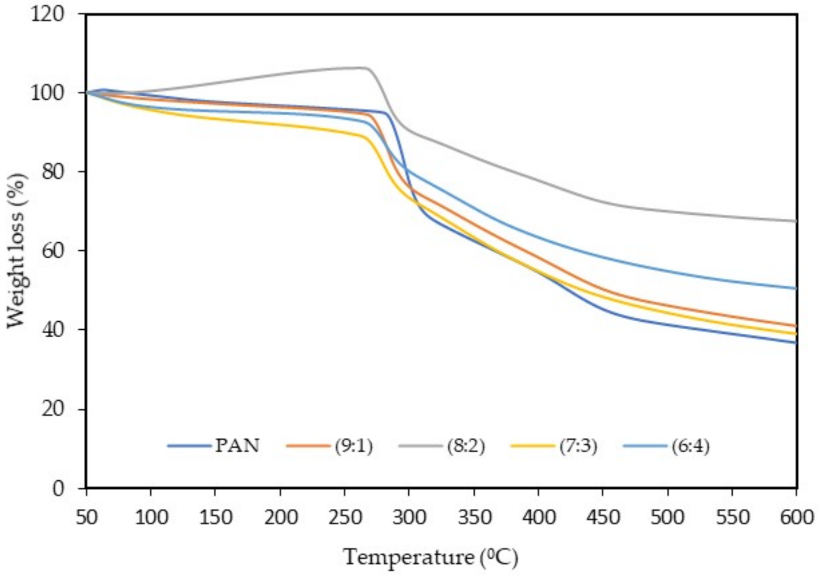

Figure S1. TGA curve of nanofibers before selective chemical dissolution.

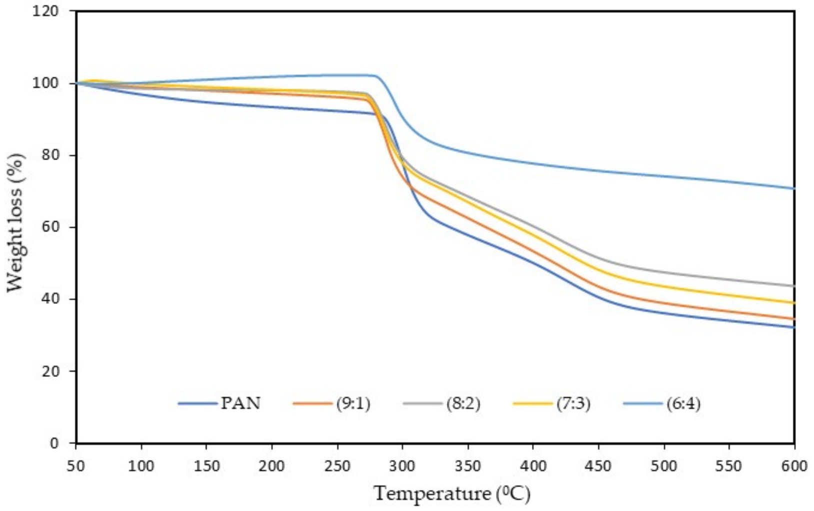

Figure S2. TGA curve of nanofibers after selective chemical dissolution.
